# Lactate kinetics in sepsis and septic shock: a review of the literature and rationale for further research

**DOI:** 10.1186/s40560-015-0105-4

**Published:** 2015-10-06

**Authors:** Jason Chertoff, Michael Chisum, Bryan Garcia, Jorge Lascano

**Affiliations:** Department of Medicine, University of Florida College of Medicine, 1600 SW Archer Road, Gainesville, FL 32608 USA; Division of Pulmonary, Allergy, and Critical Care, University of Alabama-Birmingham, Birmingham, AL USA; Division of Pulmonary Critical Care, University of Florida, Gainesville, FL USA

**Keywords:** Lactate, Kinetics, Sepsis, Early sepsis, Late sepsis, Resuscitation, Microvascular, Shock

## Abstract

Over the last two decades, there have been vast improvements in sepsis-related outcomes, largely resulting from the widespread adoption of aggressive fluid resuscitation and infection control. With increased understanding of the pathophysiology of sepsis, novel diagnostics and resuscitative interventions are being discovered. In recent years, few diagnostic tests like lactate have engendered more attention and research in the sepsis arena. Studies highlighting lactate’s prognostic potential for mortality and other outcomes are ubiquitous and largely focus on the early stage of sepsis management, defined as the initial 6 h and widely referred to as the “golden hours.” Additional investigations, although more representative of surgical and trauma patients, suggest that lactate measurements beyond 24 h from the initiation of resuscitation continue to have predictive and prognostic utility. This review summarizes the current research and evidence regarding lactate’s utility as a prognosticator of clinical outcomes in both early and late sepsis management, defines the mechanism of lactate production and clearance, and identifies areas warranting further research.

## Introduction

Nearly a decade since the landmark article, “Surviving Sepsis Campaign Guidelines for Management of Severe Sepsis and Septic Shock,” sepsis remains a hotbed of research, and new diagnostic and resuscitative interventions are under continual development and evaluation. One such effort that has received focus is the role of lactate monitoring and whether measurements taken on initial presentation and serially can be used as targets of clinical end points including adequate resuscitation and in-hospital mortality [1–6].

## Review

### Mechanism of hyperlactemia in sepsis and septic shock

Traditionally, lactic acidosis in sepsis is attributed to anaerobic glycolysis due to inadequate oxygen delivery. However, it has become clear that the mechanism of hyperlactemia in sepsis is multifactorial and due to factors beyond hypoxic tissue injury alone [5–9]. For example, James et al. proposed that lactic acidosis refractory to standard resuscitation is frequently due to increased aerobic glycolysis in skeletal muscle secondary to epinephrine-stimulated Na^+^, K^+^-ATPase activity and not to anaerobic glycolysis from hypoperfusion, and warned that continued attempts at resuscitation targeting lactate levels could lead to unnecessary blood transfusion and use of inotropic agents [8]. Furthermore, Gutierrez et al. emphasized that the etiology of prolonged lactic acidosis in sepsis is often multifactorial, making it an unreliable marker of oxygen debt and inadequate resuscitation [7]. Interestingly, it has been demonstrated that septic patients with hyperlactemia after 24 h of resuscitation had similar lactate production but lower lactate clearance than septic patients with normal blood lactate [9]. This finding raises doubts about the reliability of hyperlactemia as an indicator of the intensity of anaerobic metabolism in septic patients, suggesting instead that persistence of hyperlactemia during sepsis may be more representative of inadequate lactate clearance as opposed to pure lactate overproduction [9].

These findings questioned the notion that hyperlactemia in sepsis and septic shock is solely the result of hypoperfusion. Further support for the role of inadequate lactate clearance stems from Gibot et al. who used an endotoxemia model to demonstrate that lactate levels in sepsis can be elevated despite adequate systemic perfusion, blood pressure, and oxygen delivery [10]. Similarly, in a pig model, Ven Genderen et al. showed that septic shock behaves differently from obstructive/circulatory shock that even when cardiac output and other systemic parameters are optimized, there continues to be regional microvascular oxygen mismatch in septic shock, as compared to obstructive/circulatory shock. The authors postulate that this regional microvascular oxygen mismatch leads to elevations in lactate that may be partially unresponsive to traditional resuscitative interventions that only target systemic parameters [11]. Likewise, Hernandez et al. showed lactate in septic shock to have a biphasic response, with an initial rapid improvement followed by a much slower normalization many hours later, and hypothesized that this slow and delayed response might be attributed to the microvascular oxygen mismatch as described by Ven Genderen et al., making further systemic resuscitation through traditional sepsis resuscitation bundles ineffective and perhaps detrimental [12]. In fact, Marik et al. attribute hyperlactemia in the later phase of sepsis to increased aerobic glycolysis due to a stress response and also label any attempts at using traditional sepsis therapies to normalize lactate during this later stage as flawed and potentially harmful [13]. With these findings, Rivers et al. warn against using only lactate clearance as a marker of sepsis recovery and state that lactate clearance, central venous oxygen saturation (ScvO_2_), and other markers are complementary and not mutually exclusive end points [14].

Thus, new insight regarding the mechanism for hyperlactemia in sepsis following adequate initial resuscitation and infection control demonstrates that tissue hypoxia is not the sole etiology of hyperlactemia during late sepsis. Nevertheless, data supporting the clinical utility of lactate as a marker of early sepsis recovery is robust while the role of continued lactate monitoring beyond the initial resuscitation period into the stage of late sepsis and its potential to guide treatment during this later stage remains uncertain [15–18].

### Lactate as a prognosticator in early sepsis management

In their 2004 and 2008 sepsis guidelines, Dellinger et al. recommended measurement of lactate on initial presentation, with an elevated value signifying tissue hypoperfusion and necessitating aggressive resuscitation [19–21]. Although their guidelines suggested measuring lactate only upon presentation, many clinicians and researchers have attempted to capitalize on the test’s theoretical diagnostic and predictive value by including additional measurements during the resuscitation process [22–26]. For example, it was shown that lactate clearance greater than 10 % from initial measurement during the first 2 to 6 h of resuscitation predicted survival from septic shock and that protocols targeting lactate clearance of at least 10 % produced similar short-term survival rates to protocols using ScvO_2_ monitoring [2, 22, 23, 25]. Moreover, it was demonstrated that for every 10 % increase in lactate clearance, there was a corresponding 11 % decrease in in-hospital mortality [2]. Similarly, septic patients with lactate clearance of greater than 20 % during the initial 8 h of resuscitation had a 22 % decline in the relative risk of mortality, compared with patients having lactate clearances of less than 20 % [24].

Since these initial studies are evaluating lactate as a marker of recovery in sepsis and septic shock, further research has evaluated the role of lactate monitoring during the early resuscitative period. For example, Puskarich et al. studied resuscitation during the initial 6 h of treatment and demonstrated that achieving an ScvO_2_ goal ≥70 % without obtaining a lactate clearance goal ≥10 % was associated with higher mortality than reaching the lactate clearance goal without the ScvO_2_ goal [27]. Furthermore, these same authors showed that early lactate normalization (within 6 h) was a predictor of survival in patients being treated for sepsis and septic shock [28]. Nguyen et al. investigated the addition of lactate clearance within the first 12 h of resuscitation to the severe sepsis resuscitation bundle and showed that including lactate clearance leads to an almost twofold increase in relative risk reduction of death [29]. In response to the convincing literature supporting the utility of lactate clearance in early sepsis, the newest surviving sepsis guidelines for early goal-directed therapy (EGDT) now includes lactate clearance during the first 6 h of resuscitation as a goal of early resuscitation [24]. Thus, research regarding lactate monitoring as a marker of recovery in severe sepsis and septic shock has proven fruitful but has primarily focused on the early resuscitation period [23–28].

### Lactate as a late prognosticator in sepsis management

Literature evaluating the clinical and predictive value of lactate measurements beyond the initial 6-h resuscitation period in the medical management of sepsis is significantly less robust [30–34]. In a study of 137 surgical intensive care unit (SICU) patients, Husain et al. showed elevated initial and 24-h lactate levels to be significant predictors of mortality, with mortality ranging from 10 to 67 % depending on whether lactate levels normalized or failed to normalize at 24 h, respectively [35]. In another study investigating SICU patients, Bakker et al. showed that lactate clearance measured 24 h after admission was a significant predictor of in-hospital mortality and that the duration of persistent lactic acidosis was more predictive of mortality than the initial lactate value [30]. Similarly, Friedman et al. showed in a 35-patient study that survivors of severe sepsis admitted to the medical intensive care unit (MICU) or SICU had significantly lower lactate values at 24 h of resuscitation than nonsurvivors [31]. Finally, Manikis et al. followed lactate measurements every 8 h for >72 h in 129 trauma patients and demonstrated serial lactate measurements and the duration of hyperlactemia to be reliable indicators of morbidity and mortality following trauma [32–34]. In a 94-patient SICU sepsis study, Marty et al. measured lactate at time_0_ (*T*), *T*_6_, *T*_12_, and *T*_24_ and showed that the best predictor of death was the *T*_24_ clearance. These authors concluded that during the first 24 h in the ICU, hyperlactemia, even after the “golden hours,” is associated with increased mortality, and lactate clearance-directed therapy should be considered for the first 24 h of treatment [36]. Similarly, in an 81-patient study, Herwanto et al. investigated the role of 6-, 12-, and 24-h lactate clearance in patients with sepsis and septic shock and found only the 24-h lactate clearance measurements to be associated with mortality [37].

### Rationale for further research

Monitoring serial lactate measurements during early sepsis is a clinically useful tool as both a clinical end point to target and for prognostication; however, the utility of monitoring lactate beyond the initial 24 h of treatment is a poorly studied topic in the current literature.

Although late sepsis is loosely defined in prior studies, it is characterized by microcirculatory dysfunction leading to end organ failure and increased mortality [11, 12, 15, 38, 39]. Interestingly, failure to achieve lactate clearance beyond the golden hours despite microcirculatory restoration suggests that normalization of this microcirculatory dysfunction likely requires therapeutic interventions that differ from those used in early sepsis management [38, 40, 41]. Moreover, after the initial resuscitation has lead to restoration of microcirculatory parameters (i.e., cardiac output, ScvO_2_, mean arterial pressure), further attempts at normalizing lactate with traditional resuscitative techniques are likely detrimental [11, 12, 15]. For these reasons, further research evaluating the role of lactate kinetics during late sepsis management has the potential to guide physicians in their management of septic patients and improve clinical outcomes. Should an association between poor lactate kinetics and mortality exists during this later period, further studies could then investigate interventions targeting the microcirculatory impairment among septic patients with abnormal lactate kinetics after the first 24 h of resuscitation [38, 39].

Recently, the ProCESS, ARISE, and ProMISe trials have provided convincing evidence that EGDT is not superior to usual care in sepsis management [42–44]. With the foundational treatment paradigm for sepsis now being called into question, it is crucial to investigate the benefits of the individual components of sepsis management, both early and later in the course of care. Studies attempting to elucidate the potential value of one particular sepsis prognostic indicator, specifically lactate, later in the management of septic patients could prove beneficial [45].

## Conclusions

Extensive investigations into the efficacy of lactate kinetics as a marker for response to resuscitative therapies in septic patients have demonstrated a clear association with clinical outcomes including mortality. However, the majority of these studies have focused on the utility during the early phase of sepsis management, with little attention regarding later time periods. Future studies focusing on lactate as a potential prognostic tool for late sepsis management and treatment duration have the potential to direct physicians in their care for septic patients during this understudied time period and improve patient outcomes.
